# Irrigation of Olives with Reclaimed Wastewaters and Deficit Strategies Affect Pathogenic Bacteria Contamination of Water and Soil

**DOI:** 10.3390/pathogens11050488

**Published:** 2022-04-20

**Authors:** Gaetano Alessandro Vivaldi, Salvatore Camposeo, Gabriele Caponio, Giuseppe Lopriore, Francesco Discipio, Francesca Apollonio, Francesco Triggiano, Osvalda De Giglio, Maria Teresa Montagna

**Affiliations:** 1 Department of Agricultural and Environmental Science, University of Bari Aldo Moro, Via Amendola 165/A, 70126 Bari, Italy; gaetano.vivaldi@uniba.it (G.A.V.); salvatore.camposeo@uniba.it (S.C.); gabriele.caponio@uniba.it (G.C.); francescodiscipio@libero.it (F.D.); 2 Department of Science of Agriculture, Food and Environment, University of Foggia, Via Napoli 25, 71122 Foggia, Italy; giuseppe.lopriore@unifg.it; 3 Interdisciplinary Department of Medicine, University of Bari Aldo Moro, Piazza G. Cesare 11, 70124 Bari, Italy; francesca.apollonio@uniba.it (F.A.); francesco.triggiano@uniba.it (F.T.); mariateresa.montagna@uniba.it (M.T.M.)

**Keywords:** DESERT technology, regulated deficit irrigation, *Escherichia coli*, enterococci, sulfite-reducing Clostridia, *Salmonella* spp.

## Abstract

This study aimed to evaluate pathogenic bacterial contamination of the water-soil-plant system in potted olive trees irrigated with reclaimed wastewater. Desalinated water (DW) obtained by treating municipal wastewater (SW) and reclaimed water (RW) obtained by mixing SW with the brine (BR) produced by DESERT technology (tertiary treatment by ultrafiltration, active carbon and reverse osmosis) were used. Two different irrigation regimes were compared: full irrigation (FI) and regulated deficit irrigation (RDI). During two irrigation seasons the concentrations of *Escherichia coli*, enterococci, spores of sulfite-reducing Clostridia (SRC) and *Salmonella* spp. were monitored in water, soil and fruit samples. Microbial concentrations in DW were always below the threshold for reuse in agriculture, while RW showed the highest level of contamination for all observed parameters. RDI management appeared to increase the soil content of SRC spores with respect to FI. Sporadically low SRC spore contamination was recorded in some fruits only in 2018, regardless of the irrigation source, probably because of accidental contamination during sampling or post-harvest handling. This study encourages the creation of a better regulatory framework reference, with specific guidelines for the use of RW as part of integrated environmental systems for the management of sustainable agriculture.

## 1. Introduction

Climate change affects health determinants such as air quality, drinking water safety and food availability. Pathologies and deaths caused by the increase in temperature, extreme events and altered and/or polluted ecosystems represent significant problems, which may have negative consequences in economic terms and for the health of the population [1]. Moreover, the availability of water resources, closely connected to climate change, is undoubtedly one of the most critical problems to be faced worldwide.

During the next 25 years, droughts will increase in arid and semi-arid areas, and water will be an increasingly valuable asset [2]. A United Nations report pointed out that, in 2018, 2.3 billion people lived in water-stressed countries, of which 721 million lived in highly and critically water-stressed countries [3]. An increase in groundwater extraction will not be possible because groundwater is already over-depleted, particularly in coastal areas that are experiencing salinization from seawater intrusion [4]. Research also indicates that food production is expected to increase by around 70% globally by 2050 and by nearly 100% for developing countries, increasing the demand for water [5]. Italy is considered a medium-high water-stressed country because it uses, on average, approximately 35% of its renewable water resources. Water scarcity occurs mainly in southern areas, with critical peaks in summer due to low rainfall combined with increasing demand, correlated with the increase in tourism, agriculture and animal husbandry [6].

Globally, treated wastewater is used as an alternative water source for agricultural irrigation, with different regulations and limits in different countries [7]. Wastewater application can affect the complex water-soil-crop system, as nutrient availabilities and uptake [8] and fruit quality are affected by providing additional nutrients [9]. Advanced treatment is thus needed when producing reclaimed water (RW) to avoid a negative impact from salinity levels on both soil and crop and on public health from the presence of pathogenic infectious agents, which affect humans and/or animals [7]. Although agricultural irrigation with RW brings several advantages, various risks are related to the reuse of urban wastewater in agriculture. From the point of view of chemical-physical characteristics, urban wastewater is made up of a very diluted and weak alkaline solution, containing organic and inorganic substances, suspended solids and colloidal dispersions. This source contains approximately 99.9% water and 0.02–0.03% suspended solids, with the remainder comprising other insoluble organic and inorganic substances [7].

Some risk factors (e.g., microbial pathogens) become evident in a short period of time owing to the potential probability of human, animal or environmental contact; others become dangerous in the long term because they tend to increase as recycled water continues in use (e.g., increasing salinity can affect crop growth and soil structure). However, numerous studies [10,11,12,13] have confirmed that microbiological contamination remains a critical problem to be resolved in the reclamation of municipal wastewater (SW) for agriculture.

Possible pathogens resistant to disinfection treatments can be transferred to soil and plants by irrigation. These can represent a potential health risk not only for operators but also for consumers as the pathogens enter the food chain [14,15]. Numerous studies have indicated that a large number of microorganisms, including human pathogens (i.e., bacteria, viruses and parasites) can be found in recycled water, invade plant tissues through the root, translocate and survive within leaf crops [16,17,18].

A wide range of bacteria can be detected in wastewater. Because the pathogens of fecal origin are numerous and the presence of certain pathogens is random in time and space, water quality is normally assessed by fecal contamination indicators [19,20]. The level of microbial pollution of urban wastewater is assessed through the search for universally recognized indices of fecal contamination such as total coliforms, *Escherichia coli*, enterococci, *Salmonella* spp. and spores of sulfite-reducing Clostridia (SRC). However, national and international legislation establishes a limit for wastewater for irrigation purposes only for *E. coli* and *Salmonella* spp. [21,22].

*E. coli* is considered to be a reliable, specific indicator of fecal contamination, and is adopted with *Salmonella* in most European regulations as a basic tool for evaluating the microbiological quality of treated urban wastewater. The *Salmonella* genus includes bacteria that are widely distributed in nature. These are pathogenic to humans, especially when fruit and vegetables are eaten raw, so their presence in treated water is not permitted [18,23,24]. These microorganisms survive for weeks in waste and surface water and can easily adapt to extreme environmental conditions [25]. Enterococci can be recovered from vegetations and surface water due to contamination by animal excreta, untreated sewage and poorly treated wastewater [26,27,28]. Enterococci, compared with *E. coli*, have greater resistance in the environment and are also detectable in soil [29,30,31].

Another valid microbiological parameter for determining the quality of environmental matrices in which other fecal contamination indicators are more easily eliminated is the spores of sulfite-reducing Clostridia (SRC) [27]. In particular, *C. perfringens* appears to bemore resistant in the environment and to disinfection treatments than coliforms and enterococci and it has been isolated from sewage and marine sediments [27,32].

Agriculture remains the largest wastewater consumer because it is a sector that is significantly affected by water scarcity and is one economic sector in which wastewater use is of real benefit [33]. There are considerable variations in global legislative approaches to establishing acceptable contamination levels of wastewater reuse in agriculture. The World Health Organization (WHO) has provided relatively flexible guidelines [34] using empirical epidemiological evidence, but many countries have preferred to adopt more restrictive internal regulations. Italian legislation indicates wide availability towards the reuse of wastewater on agricultural soil [21] but, at the same time, imposes extremely rigid microbiological limits (10 CFU/100 mL of *E. coli* in 80% of the samples and a maximum of 100 CFU/100 mL as a peak value, and absence of *Salmonella* spp.).

Numerous studies have been conducted on the use of urban wastewater to irrigate different fruit tree species, such as vineyards [35], *Citrus* spp. [36], nectarines [37,38], almonds [39,40] and olive [41,42]. Olive trees provide the most representative fruit tree crop of the Mediterranean basin, can produce approximately 80% of the world’s olive oil and are appropriate candidates for the reuse of recycled water [8]. Water consumption for olive growing is strongly influenced by the cropping system, as well as by the irrigation strategy used [43]. There are few studies into estimation of exposure to microbiological pollutants at population levels [44].

This study aimed to evaluate the microbiological impact on the water-soil-tree system of potted olive trees irrigated with two different qualities of reclaimed wastewater, combined with two different systems of irrigation management. It has generated data that allows an assessment of the consequences of applying RW resources in agriculture, with particular emphasis on the risks to people and the environment of microbiological pollution.

## 2. Results

The highest monthly maximum temperatures were observed during June (average value = 29.9 °C), July (average value = 31.9 °C) and August (average value = 32.8 °C) 2017. In the same irrigation season (from May to October), the rainfall in 2017 (average value = 123.2 mm) was almost 100 mm lower than that observed in 2018 (average value = 215.6 mm).

### 2.1. Microbiological Contamination of the Treated Water

Table 1 and Table 2 show the results of microbiological monitoring of the different types of treated water during the 2017 and 2018 irrigation seasons.

SW showed the highest values of *E. coli*, enterococci and SRC spores, while treatment eliminated *E. coli* and enterococci in desalinated water (DW) (Table 1). After treatment in DW, there was a reduction in levels of SRC spores, although they were still present in only one sample in 2017 and 2018, respectively, and in low quantities (Table 2). BR showed similar values to DW, while RW, although showing high levels of contamination, had lower levels than SW because mixing with water of high salt content (BR) allowed a reduction in the microbial load. *Salmonella* spp. were always absent in all water samples (Table 2).

### 2.2. Microbiological Contamination of Soil

*E. coli* contamination was detected in DW-FI (Full Irrigation) and in larger quantities in RW-FI in 2017. In 2018, this was considered to be irrelevant because it was found at very low levels only in one of four samples from RW-FI (Table 3).

**Figure 1:**
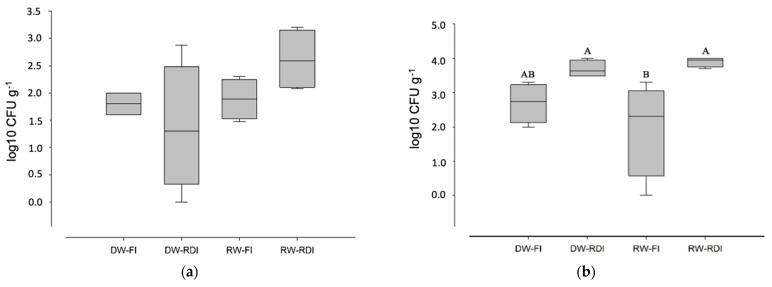
Concentrations of sulfite-reducing Clostridium spores in soil (Log CFU g^-1^) managed with different water sources (DW and RW) and irrigation strategies (FI and RDI) in 2017 (**a**) and 2018 (**b**). DW (desalinated water), RW (reclaimed water), FI (full irrigation), RDI (regulated deficit irrigation). Upper-case letters represent significant differences among treatments (*p* < 0.05).

Enterococci were detected in low quantities in all treatments in 2017 (Table 3). *Salmonella* spp. were always absent. It was observed that a regulated deficit irrigation (RDI) strategy generally had a positive effect in reducing *E. coli* and enterococci contamination in the soil. Figure 1 shows the results relating to SRC spore contamination in the soil. These spores were detected in all soil samples analyzed, confirming that they were always present in RW and DW, even if in different quantities (Figure 1). The quantities detected in 2018 were on average higher than those detected in 2017, probably because of accumulation over time (Figure 1). Statistical differences were not detected in 2017 (*p*-value Kruskal–Wallis test: 0.08166). RDI management seemed to increase the soil content of SRC spores for RW and DW in 2018. In fact, RW-FI soil contamination by SRC spores in 2018 was statistically lower (mean value of 2.0 log10 CFU/g) than in soil irrigated with RW-RDI and DW-RDI (mean values of 3.9 and 3.74 log10 CFU /g, respectively).

### 2.3. Microbiological Contamination of Fruits

In 2017, only DW-FI treatment showed enterococcal contamination with a maximum of 200 CFU/g (Table 4).

In 2018, negligible clostridial contamination was recorded for DW-FI, DW-RDI and RW-FI treatments, with a maximum value of 30, 20 and 10 CFU/g, respectively. Both *E. coli* and *Salmonella* spp. were always absent regardless of FI and RDI management.

## 3. Discussion

Health hazards are associated with the inappropriate treatment and uncontrolled reuse of reclaimed wastewater. There is the risk that humans or animals will develop infectious disease from the consumption of reclaimed wastewater containing various waterborne pathogens, and the water may present an occupational hazard to field workers who come in contact with it [44,45]. Italian and some international legislation [21,22] only provides an indication of the maximum *E. coli* concentrations permitted in wastewater reused in irrigation. In our study, neither the BR produced by the DESERT prototype, nor the DW, showed *E. coli* contamination, while RW (SW + BR) showed high contamination levels. However, the mix of SW with high salt content water (BR) was able to reduce the microbial load of the water compared with SW alone as a result of a dilution effect even if it exceeded the limit imposed by the Italian legislation on the reuse of wastewater [21]. Moreover, legislation [21,22] does not provide any indication of detection and the maximum limit of enterococci and SRC spores permitted in the reuse of wastewater for fruit tree irrigation. Although enterococci and SRC spores are resistant to disinfection in SW and are able to survive for long periods in the environment [46], the DW eliminated enterococci and significantly reduced the numbers of SRC spores. Since SW always resulted in contamination by Enterococci and SRC spores, this contamination was always found in the RW samples resulting from the mixing of SW and BR.

For bacteria, the smallest of which are 0.2–0.3 µm, the use of ultrafiltration, where the pores of the membranes are smaller than 0.1 µm, should guarantee a 100% efficiency of their retention [47]. Indeed, DW derived by ultrafiltration process of SW showed only one positive sample for Enterococci and two samples for SRC spore bacteria during the entire period of study (2017–2018), both with low concentration. It is known that no membrane filtration system can be considered as an absolute barrier for all microorganisms [44]. This is primarily due to imperfections of membranes and membrane modules and the possibility of secondary bacterial growth in water after passing through the membrane [48]. For example, in another study, SRC spore bacteria was also found after ultrafiltration treatment [49]. In fact, the cells of microorganisms could penetrate the pores of the membrane with diameters much smaller than the dimensions of the cells themselves, due to pressure deformation [48].

Several studies have shown that the survival of microorganisms in the soil is a function of different properties, including texture, organic matter, moisture, irrigation regime, pH and chemical fertilizer [27,50]. These factors have an important role in the formation and stabilization of aggregates composed of sand and silt particles, which can retain enough water within them for microorganisms to survive [51]. Indeed, the soil property that appears to have the greatest impact on bacterial survival is moisture retention, which is related to the distribution of particle size and organic matter content. Soils with high clay content usually provide a more protective environment and allow enteric bacteria to survive for a long time compared with sandy or loam soils [52]. Our study considered loam soil, and we were able to confirm this evidence, as a reduction of the bacterial load was observed.

The low level of soil organic matter may produce unfavorable conditions for the survival of fecal bacteria. According to Durán et al. [53], exogenous bacterial survival is severely hampered in loamy sand and in soils with little organic matter.

Following comparison of the levels of *E. coli* and enterococci contamination of a RW water source with that of soil, a significant reduction was observed in soil, and, in general, microbial contamination in soil was very slight. An analogous performance was reported by Palese et al. [12], who found that the soil reduced fecal contamination after only 10 days from the end of the irrigation season.

The absence of *E. coli* soil contamination in 2018, despite its presence in the RW source, was higher than in 2017. The contamination of one of four DW-FI samples in 2017, despite the absence of *E. coli* in the DW source, may suggest background levels of fecal coliforms, according to Black et al. [54]. This sporadic contamination may have been due to wild animals (birds and rodents) crossing the field in question [55]. This suggestion may also explain the low enterococcal contamination occasionally found in soil samples, regardless of the treatment.

SRC spores were detected in all the soil samples analyzed, indicating the omnipresence of these bacteria [32]. The quantities found in 2018 were, on average, higher than those detected in 2017. Levels of SRC spores were particularly high in both RDI treatments, so the irrigation strategy allowed their accumulation in the upper soil layers (0–10 cm) because of the lower water volumes distributed with respect to FI treatments. This indicated that the irrigation strategy can strongly influence the vertical migration of pathogens through the soil profile, preventing or favoring subsurface transport. As a result, and in agreement with Palese et al. [12], there appears to be a need to consider SRC spores as additional fecal indicators within the guidelines for wastewater reuse. The vegetative form of bacterium *C. perfringens* is the main etiological agent of myonecreosis of connective tissues [56] and may be also responsible for food poisoning and diarrhea [57].

In neither year was contamination by *E. coli* and enterococci detected on fruits irrigated with both DW and RW (apart from one of four cases in DW-FI samples in 2017; this probably resulted from causes other than irrigation), mainly thanks to the drip irrigation method adopted. These results aligned with those of Palese et al. [12], who used the same irrigation method in an olive grove irrigated with SW.

The Italian Ministerial Decree 185/2003 [21] provides that treated wastewater used in an irrigation area takes into consideration specific irrigation methods, favoring those systems that avoid contact between the edible part of the plant and the treated wastewater and allowing a large water saving. The localized irrigation technique is the most appropriate for this purpose. In fact, drip systems—because of the low volumes of irrigation—allow significant water savings because the water is directed to the root system of the crop, increasing its efficiency and effectiveness. These systems avoid the washout of nutrients, do not wet the vegetation and therefore do not promote parasitic attacks. They also reduce the development of weeds as they wet only small portions of the soil, and so are preferred systems for irrigating olive groves.

We consider that the environmental conditions of our experiment may have influenced the level of contamination of fruits. In particular, the high levels of solar radiation and temperature during fruit ripening and the harvesting period may have destroyed pathogenic bacteria on the fruit surface. These conditions may particularly have affected those less resistant to environmental conditions such as *E. coli* and enterococci. Low rainfall during the olive ripening period also hindered the transport and dissemination of microbial agents [52]. Finally, the low SRC spore contamination in 2018 (from <10 to 30 CFU/g) recorded in fruits in four of 12 cases, regardless of the irrigation source, was probably due to environmental pollution, accidental contamination occurring during sampling or post-harvest handling [12].

## 4. Materials and Methods

### 4.1. Experimental Site and Plant Material

This study was carried out during two consecutive years (2017 and 2018) in an experimental orchard of 2-year-old (in 2017) self-rooted olive trees (cv Arbosana). Trees were transplanted in January 2017 into 100 L polyethylene pots (diameter 50 cm, height 65 cm) filled with soil. The soil texture was classified as loam (44.8% sand, 42.9% silt and 12.3% clay) [58], and the organic matter (%) was 0.91 ± 0.21. Trees were placed outdoors in a plot at the University of Bari Aldo Moro (Apulia, Italy) experimental station, located in southeastern Italy (41°06′41″ N, 16°52′57″ E, 5 m above sea level). Pots were placed on the ground in a 1.85 × 2.10 m^2^ planting system in rows-oriented N–NE to S–SW. ET_0_ was calculated with the Penman-Monteith method, and all data were provided by a climate station located approximately 100 m from the experimental platform. The monthly maximum and minimum temperature, and rainfall during the sampling periods were recorded. The same amounts of macronutrients (N–P_2_O_5_–K_2_O) were distributed in all treatments by a fertigation system. Common agronomical practices (pruning, weeding and pest control) were performed in the experiment.

### 4.2. Water Sources and Irrigation Treatments

**Figure 2:**
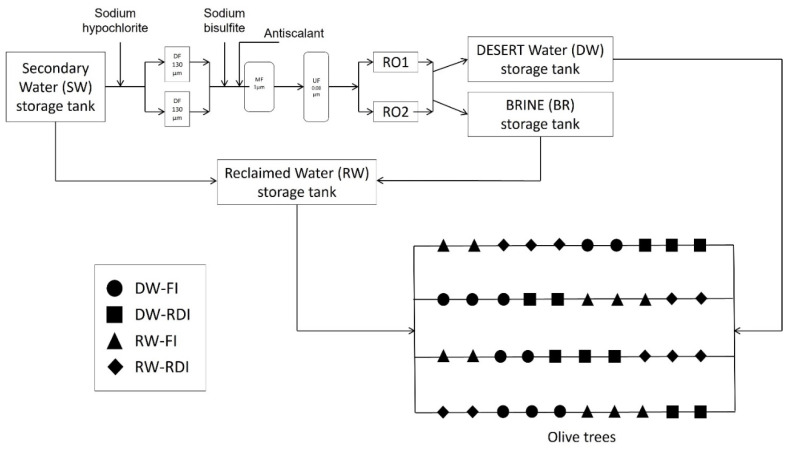
Flowchart of the SW (secondary treated water), desalinated water (DW), brine (BR), reclaimed water (RW) and the experimental test field.

Two irrigation water sources were applied: DW and RW. DW was obtained by treating SW (from the Bari-Sud secondary treatment plant) through the DESERT prototype (DEsalination and SEnsoR Technology), a tertiary treatment plant using ultrafiltration, active carbon and reverse osmosis [42,59] to reach a water electrical conductivity (ECw) of ~1 dS m^−1^. RW was obtained by mixing SW with the brine (BR) produced by the tertiary DESERT prototype until an ECw of 3 dS m^−1^ was reached (Figure 2). In particular, for each 5 m^3^ of SW, the prototype produced 1 m^3^ of BR. The proportion of mixture was variable and monitored with a continuous monitoring system of water electrical conductivity.

For each water source, two irrigation managements were applied: full irrigation (FI) and regulated deficit irrigation (RDI). FI management involved irrigation at 100% of ETc (crop evapotranspiration) during the whole irrigation season with DW or RW (DW-FI and RW-FI, respectively). RDI management consisted of irrigating at 100% ETc, except during the pit-hardening phenological phase, when the trees received one-half of the water applied during FI (50% ETc) with DW or RW (DW-RDI and RW-RDI, respectively).

The irrigation system and scheduling are described by Vivaldi et al. [39]. The irrigation season ended 2 weeks before harvest [54,60]. A total of 40 trees made up this assay (10 per treatment). Each irrigation treatment had five replicates, distributed in a completely randomized design. Each replicate consisted of two trees.

### 4.3. Water Sampling and Monitoring

From August 2017 to October 2018, 12 water samples were collected: 4 in 2017 and 8 in 2018.

Sterile containers were used for the sampling, which was performed under calm conditions with no rain, between 8:00 a.m. and 11:00 a.m. A total of 2 L of treated wastewater was collected during each sampling, transported in a refrigerator (+4 °C) and processed within 5 h. *E. coli*, enterococci and SRC spores were selected as indicators of fecal contamination, while *Salmonella* spp. were selected as pathogenic bacteria.

Specific aliquots of each water sample were filtered through a cellulose ester membrane with a diameter of 47 mm and a pore size of 0.45 µm (Millipore, Milan, Italy).

For *E. coli*, 100 mL of water sample was filtered, and the membrane was placed on plates containing Chromogenic Coliform Agar (Biolife Italiana Srl, Milan, Italy). After incubation at 36 ± 2 °C for 24 ± 2 h, the blue-violet colonies were identified as *E. coli*, and the salmon-pink, oxidase-negative colonies were identified as coliform bacteria [61]. The results were reported as CFU/100 mL, and the limit of detection (LOD) was <1 CFU/100 mL.

For enterococci, a 100 mL aliquot of each sample was filtered, and the membrane was placed over a Slanetz and Bartley agar medium (Biolife Italiana srl) and incubated at 36 ± 1 °C for 48 h. The colonies ranged in color from pink to dark red and brown, but only catalase and esculin hydrolysis-positive colonies were considered to be enterococci [62]. The results were reported as CFU/100 mL, and the detection limit was <1 CFU/100 mL.

The spores of sulfite-reducing Clostridia were determined using the APAT method [63]. Water samples (100 mL) were pretreated for 10 min at 80 °C. The spores were numbered using the membrane filter technique with SPS agar (Biolife Italiana srl). The plates were incubated for 24–48 h at 37 °C in an anaerobic jar, where the anaerobic atmosphere was generated with the Anaerogen system (Oxoid, Basingstoke, UK). Black colonies were considered to be spores of sulfite-reducing Clostridia. The results were reported as CFU/100 mL, and the detection limit was <1 CFU/100 mL.

The determination of *Salmonella* spp. was also carried out by the APAT method [63]. A 1-L sample was filtered through a cellulose membrane (47 mm Ø and 0.45 mm mesh; Millipore, Milan, Italy). The membrane was immersed in 100 mL of Buffered Peptone Water (BPW) (Merck, Darmstadt, Germany) and incubated at 36 ± 1 °C for 18–24 h. A 0.1 mL aliquot was transferred to 10 mL of Selenite Cystine Broth (Biolife Italiana srl) and incubated at 36 ± 1 °C for 24–48 h 24 h. The medium was striated after 24 h and 48 h inside XLD agar plates (Biolife Italiana srl) and Hektoen Enteric Agar (Merck). After 24 h at 37 °C, colonies with a typical morphology were streaked on Tryptic Soy Agar plates (Biolife Italiana srl), incubated at 36 ± 1 °C for 24 h and subjected to biochemical confirmation tests (API 20E, Biomèrieux, Marcy l’Etoile, France). Presumed *Salmonella* spp. colonies were subjected to specific serological tests as described in the APAT CNR IRSA manual 7080.

### 4.4. Soil and Fruit Sampling and Monitoring

Soil samples were collected at the end of each irrigation season (November 2017 and October 2018) after being treated with different water sources and irrigation strategies. For each year, DESERT water (DW) (*n* = 8) and secondary treated RW mixed with brine (BR) (*n* = 8) were used with different irrigation strategies, namely FI (*n* = 8) and RDI (*n* = 8), for a total of 16 samples/year. The samples were collected at a depth of 10 cm from the surface to prevent the water reclaimed during the irrigation season from affecting the microbiological characteristics of the soil [12]. Before microbiological analysis, the soil samples (1000 g) were sieved to 2 mm in sterile conditions to remove plant residues and mineral fraction with due caution to limit contamination and drying of the material [64].

The soil samples (100 g) were placed in sterile plastic containers and stored at +4.0 °C until microbiological analysis and, in any case, they were analyzed within 24 h of collection.

The harvesting took place on 31 October 2017 and 21 November 2018, at the appropriate ripening stage, when the detachment index had reached at least 2 N g^−1^ [65]. For each year, a total of 16 olive samples were collected: 8 samples irrigated with DW and 8 with RW mixed with BR with different irrigation strategies, namely FI and RDI, respectively. All olives were harvested manually, using sterile gloves and plastic bags, from the portion of the foliage closest to the drippers (considered to be in the worst condition in terms of potential contamination) and were stored at +4.0 °C [12]. Samples of 10 g of olives and 10 g of soil were homogenized, respectively, with 90 mL of BPW (Biolife Italiana srl) in the stomacher, a peristaltic-type device. The homogenization time (1 min) was sufficient to allow the passage of the microbial cells from the sample to the diluent, but not so long as to cause a loss of viability of the microorganisms [66].

For *E. coli*, 1 mL of soil and fruit samples in duplicate of two successive dilutions were transferred onto two sterile plates; subsequently, 15 mL of Tryptone Bile X-GLUC medium (TBX, Biokar diagnostic, Allone, France) was added to each plate. The plates were incubated at 37 °C for 4 h, followed by 18–24 h at 44 °C. β-glucuronidase split the chromogenic substrate BCIG, and the released chromophore gave rise to distinct colonies of *E. coli* (blue-green). These blue-green colonies on TBX medium (Biokar diagnostic, Allone, France) were listed without a confirmation phase after an incubation period [67] and reported as CFU/g (LOD < 10 CFU/g).

To measure enterococci, soil and fruit samples and their dilutions were inoculated onto Slanetz & Bartley medium (Biolife Italiana srl). After plate incubation (for 48 h at 37 °C), intense red or pink colonies were transferred to Bile Aesculin Azide Agar (Liofilchem srl, Roseto degli Abruzzi (TE), Italy) and incubated for 2 h at 44 °C. When any blackening of the soil occurred, the colonies were counted as enterococci and reported as CFU/g (LOD < 10 CFU/g) [12,62].

The enumeration of sulfite-reducing Clostridia spores was carried out according to the inclusion plating method in anoxic conditions [12]. To test the SRC spores, a 10-mL aliquot from the dilution of the soil and fruit sample was incubated for 15 min at 75 °C. The suspensions were rapidly cooled and further diluted in a solution of sterile extraction. From each dilution, 1 mL was inoculated onto SPS agar plates (Biolife Italiana srl) by the inclusion plating method; the plates were then incubated for 24–48 h at 37 °C in an anaerobic jar with the anaerobic atmosphere generating the Anaerogen system (Oxoid, Basingstoke, UK). The black colonies, surrounded by a dark halo and resulting negative catalases, were identified as SRC spores. The results of soil and fruit were expressed in CFU/g (LOD < 10 CFU/g) [12].

The determination of *Salmonella* spp. was carried out on 10 g of soil sample inoculated in 90 mL of BPW, while 25 g of fruit samples was reacted with 225 mL of BPW medium. After incubation at 37 °C for 18 h, the MSRV (Rappaport Vassiliadis Semi-Solid Medium Modified supplemented with Novobiocin Antimicrobic Supplement; Biolife Italiana srl) plates were inoculated with 0.3 mL of BPW culture incubated at 41.5 °C for 24 h. At the same time, 1 mL of BPW was transferred to a test tube containing 10 mL of Muller-Kauffmann Tetrathionate-Novobiocin Broth supplemented with Iodine Solution (MKTTn) (Biolife Italiana srl) and incubated at 37 °C for 24 h. After incubation, the opaque growth of the MSRV plate and the MKTTn substrate were streaked on the XLD plate containing lysine, xylose and sodium desoxycholate, and on Hektoen Enteric Agar (Merck, Darmstadt, Germany) with lactose, sucrose, sodium thiosulfate and ferric ammonium citrate. After growth on selective media, the identification of *Salmonella* spp. was carried out in the manner previously described for water samples [68].

## 5. Conclusions

Desalinated irrigation water allowed microbiological contamination below the threshold values for reclaiming wastewater for use in agriculture. In contrast, SW mixed with BR showed the highest level of bacterial contamination. When these water sources are used for irrigation, they are subjected to further filtration through the soil, which therefore assumes the role of an additional natural, free and efficient purifier. The use of RW becomes indispensable along coastal areas where the water available for irrigation is salty.

This study may promote the use of reclaimed SW, which would otherwise flow directly into the receiving water bodies, polluting natural water resources and causing environmental impacts on seas, rivers, streams and lakes. The risks cannot be ignored, however, and these include the presence of pathogens and chemical contaminants, salinity and the damage that the prolonged and unsustainable use of these water sources can cause to the soil. These drawbacks can be monitored through sustainable and effective agricultural management practices.

Additional investigation is needed to examine the possibility of removing, through different treatments and in various soil conditions, other contaminating indicators (e.g., *Giardia* cysts, *Cryptosporidium* oocysts and viruses). An improved regulatory framework reference, with specific and innovative guidelines for the use of reclaimed SW as part of integrated systems for sustainable environmental management, is also required.

## Figures and Tables

**Table 1:** Minimum and maximum concentrations of *Escherichia coli* and enterococci in the water used (CFU/100 mL) in 2017 and 2018. SW (secondary treated water), DW (desalinated water), BR (brine) and RW (reclaimed water). P/T is the positive to total samples ratio.

Water	*Escherichia coli*								Enterococci			
	Minimum (CFU/100 mL)		Maximum (CFU/100 mL)		P/T		Minimum (CFU/100 mL)		Maximum (CFU/100 mL)		P/T	
	2017	2018	2017	2018	2017	2018	2017	2018	2017	2018	2017	2018
SW	4500	2	10,000	38,000	4/4	8/8	600	<1	600	700	3/4	7/8
DW	<1	<1	<1	<1	0/4	0/8	<1	<1	<1	5	0/4	1/8
BR	<1	<1	<1	<1	0/4	0/8	2	<1	2	<1	1/4	0/8
RW	500	3	3000	11,300	4/4	8/8	100	<1	100	600	3/4	6/8

**Table 2:** Minimum and maximum concentrations of sulfite-reducing Clostridium spores and *Salmonella* spp. in the water used (CFU/100 mL) in 2017 and 2018. SW (secondary treated water), DW (desalinated water), BR (brine), RW (reclaimed water). P/T is the positive to total samples ratio.

Water	Sulfite-Reducing *Clostridium* Spores						*Salmonella* spp.					
	Minimum (CFU/100 mL)		Maximum (CFU/100 mL)		P/T		Minimum (CFU/1000 mL)		Maximum (CFU/1000 mL)		P/T	
	2017	2018	2017	2018	2017	2018	2017	2018	2017	2018	2017	2018
SW	10,000	173	10,000	5000	4/4	8/8	<1	<1	<1	<1	0/4	0/8
DW	<1	<1	100	17	1/4	1/8	<1	<1	<1	<1	0/4	0/8
BR	<1	<1	<1	<1	0/4	0/8	<1	<1	<1	<1	0/4	0/8
RW	3000	160	3000	2000	4/4	8/8	<1	<1	<1	<1	0/4	0/8

**Table 3:** Minimum and maximum concentrations of *Escherichia coli* and enterococci in the soils (CFU/g) managed with different water sources. DW (desalinated water), RW (reclaimed water), FI (full irrigation), RDI (regulated deficit irrigation), in 2017 and 2018. P/T is the positive to total samples ratio.

Water	*Escherichia coli*								Enterococci			
	Minimum (CFU/g)		Maximum (CFU/g)		P/T		Minimum (CFU/g)		Maximum (CFU/g)		P/T	
	2017	2018	2017	2018	2017	2018	2017	2018	2017	2018	2017	2018
DW-FI	<10	<10	100	<10	1/4	0/4	<10	<10	100	1	1/4	1/4
DW-RDI	<10	<10	<10	<10	0/4	0/4	<10	<10	10	4	1/4	1/4
RW-FI	<10	<10	360	1	2/4	1/4	<10	<10	20	<10	1/4	0/4
RW-RDI	<10	<10	<10	<10	0/4	0/4	<10	<10	10	<10	1/4	0/4

**Table 4:** Minimum and maximum concentrations of sulfite-reducing Clostridium spores and enterococci on olive fruits (CFU/g) irrigated with different water sources. DW (desalinated water), RW (reclaimed water), FI (full irrigation), RDI (regulated deficit irrigation), in 2017 and 2018. P/T is the positive to total samples ratio.

Water	Sulfite-Reducing *Clostridium* Spores								Enterococci			
	Minimum (CFU/g)		Maximum (CFU/g)		P/T		Minimum (CFU/g)		Maximum (CFU/g)		P/T	
	2017	2018	2017	2018	2017	2018	2017	2018	2017	2018	2017	2018
DW-FI	<10	<10	<10	30	0/4	2/4	<10	<10	200	<10	1/4	0/4
DW-RDI	<10	<10	<10	20	0/4	1/4	<10	<10	<10	<10	0/4	0/4
RW-FI	<10	<10	<10	10	0/4	1/4	<10	<10	<10	<10	0/4	0/4
RW-RDI	<10	<10	<10	<10	0/4	0/4	<10	<10	<10	<10	0/4	0/4

## Data Availability

All data generated or analyzed during this study are included in this published article.
